# DNA Damage and Repair in Human Reproductive Cells

**DOI:** 10.3390/ijms20010031

**Published:** 2018-12-21

**Authors:** Anaís García-Rodríguez, Jaime Gosálvez, Ashok Agarwal, Rosa Roy, Stephen Johnston

**Affiliations:** 1 Departamento de Biología, Universidad Autónoma de Madrid, 28049 Madrid, Spain; anais.garcia@uam.es (A.G.-R.); jaime.gosalvez@uam.es (J.G.); 2 American Center for Reproductive Medicine, Cleveland Clinic, Cleveland, OH 44195, USA; agarwaa@ccf.org; 3 School of Agriculture and Food Sciences, University of Queensland, Gatton, QLD 4343, Australia

**Keywords:** spermatozoon, oocyte, DNA damage, DNA repair, protamine, genetics, infertility

## Abstract

The fundamental underlying paradigm of sexual reproduction is the production of male and female gametes of sufficient genetic difference and quality that, following syngamy, they result in embryos with genomic potential to allow for future adaptive change and the ability to respond to selective pressure. The fusion of dissimilar gametes resulting in the formation of a normal and viable embryo is known as anisogamy, and is concomitant with precise structural, physiological, and molecular control of gamete function for species survival. However, along the reproductive life cycle of all organisms, both male and female gametes can be exposed to an array of “stressors” that may adversely affect the composition and biological integrity of their proteins, lipids and nucleic acids, that may consequently compromise their capacity to produce normal embryos. The aim of this review is to highlight gamete genome organization, differences in the chronology of gamete production between the male and female, the inherent DNA protective mechanisms in these reproductive cells, the aetiology of DNA damage in germ cells, and the remarkable DNA repair mechanisms, pre- and post-syngamy, that function to maintain genome integrity.

## 1. Introduction

The role of sexual reproduction in conferring the potential for adaptive changes in a population relies on the phenomenon of anisogamy, or the fusion of dissimilar gametes. Anisogamy, resulting in the production of a normal zygote, provides the phenotypic variation on which natural selection may operate and is, therefore, the basis of evolutionary potential. In humans, primordial germ cells (PGCs) originate from the epiblast between eight and 14 cell divisions after fertilization. After differentiation, PGCs begin to proliferate, however, the chronology and outcome of this process is different depending on gender.

In the male embryo, PGCs expand to form a pool of spermatogonial stem cells that remain in mitotic and meiotic arrest until puberty. On reaching puberty, some of these spermatogonial stem cells enter the spermatogenic cycle on their ultimate journey to become mature spermatozoa, while others continue as stem cells throughout the life of the male. Spermatogenesis can be divided into three sequential steps, [1] (i) mitotic proliferation (spermatocytogenesis) resulting in the production of large numbers of spermatocytes, (ii) meiotic recombination and chromosome segregation producing genetically diverse haploid spermatids, and (iii) cytodifferentiation of the spermatids (spermiogenesis) involving complex morphological and genome remodeling that radically transforms the round spermatid into highly specialized, and species-specific spermatozoa. Although the quality and quantity of sperm production in a healthy male may vary post-puberty, most males have the capacity to produce spermatozoa even in their old age. In fact, it has been estimated that average male produces over 525 billion sperm in his lifetime.

Gamete production in female is substantially different; PGCs migrate into the embryonic gonad and proliferate to form a resident population of primary oocytes that remain in meiotic arrest in prophase 1 until the female reaches puberty. Following commencement of her first menstrual cycle, oocytes are periodically released from the follicular pool, and under the appropriate endocrine control, a proportion of oocytes are recruited, then selected, until typically, one becomes dominant and recommences meiosis 1. Henceforth, ovulation and fertilization with the male gamete follows, after which meiosis 2 is finalized. Although the human ovary contains approximately 1–2 million oocytes at birth, by the time a woman reaches puberty, this number reduces to 300,000 by puberty, 25,000 by the age of 37 and 0 by menopause. Thus, it has been estimated that approximately 500 mature oocytes are ovulated during the female’s reproductive lifetime, with the vast majority of gametes subjected to atresia. 

This fundamental difference in gamete production is critical to understanding the susceptibility of male and female gametes to DNA damage and the DNA repair mechanisms inherent in the spermatozoon and oocyte. This review will focus on irreparable and repairable DNA damage which is produced in human reproductive cells and the corresponding repercussions on infertility. Additionally, DNA damage response mechanisms in the different developmental phases of the spermatozoon, oocyte and zygote to conserve the genome integrity will also be reviewed. 

## 2. Genome Organization and Protection in Reproductive Cells

### 2.1. Gamete Genome Organization: Sperm Protamination

In mammals, the male gamete is the only cell that is biologically prepared for an autonomous subsistence before fertilization is accomplished, whereas the female gamete is considered a “quasi-sedentary” gamete. The oocyte is also protected by the female soma which includes the cumulus oophorus, granulosa cells and the ovarian environment, all of which help to control and regulate the maturation process. Nevertheless, there is one overwhelming difference between the spermatozoon and the oocyte regarding their chromatin organization. While the oocyte has DNA packaged into histone-like somatic cell proteins, the spermatozoon experiences a remarkable reorganization of its nucleus in the last phases of spermatogenesis, during which approximately 80% of the original histones are replaced by transition nuclear proteins (TNP) and protamines [2,3,4,5].

Protamines are small basic proteins that in eutherian mammals, including humans, contain an arginine rich core and a high concentration of cysteine residues. The arginine rich core (48% in humans) provides the protamines with a positive charge that allows them to bind tightly to the DNA which is negatively charged [6]. The cysteine residues allow disulfide bridges to form between protamine residues, facilitating both intra- and inter-protamine bonding, and consequently increasing the condensation level and stability of the nucleus [7,8]. In eutherian mammals there are two types of protamines, protamine 1 and protamine 2, although the latter is expressed only in the human and mouse. In other species, such as boars or bulls, the production of protamine 2 appears to have been abolished and only protamine 1 orchestrates DNA compactness [2]. Both protamines and the transition nuclear protein 2 are encoded together in a gene cluster contained in a DNA loop and surrounded by two “cysteine” regulatory units. This cluster is potentiated in the late pachytene stage of spermatocytes and transcribed in round spermatids, where the resulting RNAs are stored in translationally repressed ribonucleoproteins [9]. 

During the early stages of spermiogenesis, somatic histones in elongating spermatids are hyper-acetylated and modified, and the characteristic somatic nucleosomes are disassembled. Subsequently, somatic histones are replaced by TNPs. Protamines are then synthesized and phosphorylated which quickly replace the TNPs. After the protamines are bound to the DNA, they are subsequently dephosphorylated except for some specific residues (for example serine 8 and 10 in protamine 1 and serine 14 in protamine 2 for humans) [10]. Finally, during the last stages of spermiogenesis, spermiation and transit through the epididymis, intra- and intermolecular disulfide bridges are formed, allowing the DNA to further condense and stabilize [2]. The condensation of the DNA with protamines instead of histones gives the sperm cell some unique characteristics when compared to somatic cells [8] and this is no doubt related to the fact that the spermatozoon is biologically prepared for a short autonomous (ex soma) subsistence before fertilization is accomplished. This singular purpose of the sperm cell is reflected in its peculiarity and there are substantial contributions of protamines to this process, such as: (i) DNA condensation to achieve a smaller and more hydrodynamic nucleus to facilitate sperm movement and transport; (ii) extra DNA protection and stability against the negative effects of external agents such as free radicals or radiations: (iii) competition with other transcriptional factors to eliminate some of the somatic epigenetic information from the sperm nucleus, leaving it free to be reprogrammed by the oocyte after fertilization; (iv) paternal imprinting; (v) a check point in spermiogenesis (defects in protamination act as a check point activating apoptotic pathways in the spermatozoon); and (vi) post-fertilization functions in the oocyte [3].

### 2.2. Genome Domain Protection to Spontaneous Mutations 

The human genome contains approximately 3.2 billion base pairs (bp), and within every genome, functional and non-functional DNA sequences are determined by A, T, C, and G nucleotide arrangements [11]. However, genome organization is not homogeneous, and one of the most intriguing and unexplained pieces of evidence is the varying proportion of non-repetitive versus repetitive DNA sequences found in the whole genome of the most highly evolved species [12,13]. In general, protein and RNA-coding genes are non-repetitive DNA sequences that account for most of the so-called structural genes. The rest of the genome is formed by intergenic DNA sequences comprising satellite DNAs, long and short interspersed nuclear elements (LINES and SINES), long terminal repeats (LTR) or DNA transposons, all of which are assumed to have low levels of transcription. Within this distribution of genome domains, the probability that a structural gene can be affected by a single mutation is much lower than those affecting the rest of the genome. In some sense, the whole genome acts as a buffer against the putative negative effects of mutations affecting a single gene sequence. This possibility opens up a new question about heterogeneity for genome organization and mutation sensitivity for different genome domains. Experimental evidence shows that different genome domains may present different levels of susceptibility to DNA damage when exposed to equivalent external insults [14].

The DNA present in the telomeres (TEL-DNA) is highly susceptible to DNA damage [15]. Within the germ line, DNA damage affecting telomeres still requires further research. Large variations in the copy number of TEL-DNA sequences among male and female gametes are not expected, since this would produce a large heterozygosity between the homologous chromosomes. Several studies have concluded that telomerase activity and telomere length are inversely correlated in germ cells [16]. Telomerase activity is likely to be maximal in spermatogonia and oogonia and it progressively decreases throughout spermatogenesis and oogenesis, finally to be very low in the mature spermatozoon and oocyte. An opposite situation occurs for telomere length; after fertilization, the presence of critically shortened telomeres, either from the sperm or the oocyte, may contribute to abnormal cleavage and development. Despite this phenomenon, telomere lengthening has been recorded during the early cleavage cycles through a recombination-based mechanism and telomerase activity [17]. It is also worth mentioning that ejaculated spermatozoa are also not homogeneous in terms of telomere size [18] and that the routine sperm selection techniques used in assisted reproduction technologies (ART), such as density gradient centrifugation and swim-up, unintentionally allow spermatozoa with the largest TEL-DNA repeats to be selected [19,20] which, as mentioned previously, is beneficial for better embryonic development.

### 2.3. Genetic Flaws Affecting Gamete Functionality and Fertility

Approximately 15% of male and 10% of female infertility problems are related to genetic abnormalities. Assisted reproduction technologies, especially intracytoplasmic sperm injection (ICSI), have been instrumental to overcome some of these scenarios. However, with the use of these practices, some of the natural selection barriers for fertilization are bypassed, increasing the risk of transmitting unknown genetically defective parental genomes [21]. Consequently, gamete identification of genetic factors related to infertility should be a part of the standard workup of the infertile couple [22].

#### 2.3.1. Genetics and Male Infertility

Genome rearrangements mainly include incorrect chromosome numbers, chromosome rearrangements such as inversions, chromosome duplications and a combination of different chromosome mutations. These genome rearrangements occur in approximately 5% of infertile men [23] and are likely to produce unbalanced haploid gametes after meiosis. The most common chromosomal abnormality found in infertile men is XXY Klinefelter´s syndrome. This syndrome can appear both as a non-mosaic 47 XXY or as a mosaic 47XXY/46XY, and in both cases, it is related with different grades of oligo or astenozoospermia [24]. Moreover, both forms are related to an increase in the number of aneuploid spermatozoa in the ejaculate, thus increasing the risk of fathering offspring with chromosomal abnormalities such us 47XXY and 47XXX. With the use of ICSI, however, an increased number of healthy children have been born from men with Klinefelter´s syndrome [25,26]. Other chromosomal abnormalities that are more common in infertile men include 47XYY and 46XX [27]. For 47XYY, the effect on fertility may range from azoospermia to normozoospermia, whereas 46XX males are generally azoospermic due to the lack of the long arm of the Y chromosome [28].

Robertsonian translocations occur when 2 acrocentric chromosomes fuse and consequently lose part of their short arms. Although these types of translocations are rare, they are 9 times more likely to occur in infertile men than in fertile ones [29]. Reciprocal translocation is a mutual exchange of chromosome segments between non-homologous chromosomes. Although they are not generally pathological for the carrier, they have been associated with a higher incidence of infertility, especially in heterozygous individuals, where the multivalent formed at meiosis do not generally produce alternate chromosome orientation at metaphase I [30]. 

Extreme cases of genomic rearrangement are also known as complex chromosome rearrangements (CCRs), which are structural aberrations involving at least three chromosomes with three or more chromosomal breakpoints [31]. Although the incidence of CCRs in the human population is extremely low, they have been repeatedly associated with infertility, spontaneous abortions and malformations in offspring due to complications in the segregation of the derivative chromosome and meiotic failures [32,33]. Moreover, the simultaneous formation of multiple genomic rearrangements in a single event can also occur; this situation, known as chromothripsis, occurs predominantly in the paternal germ line and is often associated with developmental disorders [34,35].

**Figure 1:**
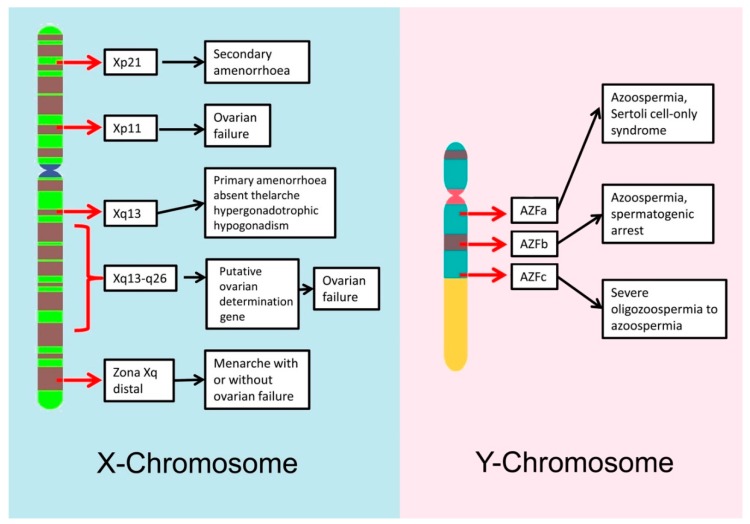
Schematic overview of the main regions at the sex chromosomes where microdeletions are directly related with infertility.

Y chromosome microdeletions are chromosomal deletions that cover several genes but are not big enough to be detected using conventional cytogenetic methods. Several studies have demonstrated that microdeletions are more common in oligo- and azoospermic men, than in normal fertile men [36,37]. The most relevant microdeletions for male fertility are those located on the long arm of the Y chromosome (Yq); there is a region on the Y chromosome known as the azoospermia factor (AZF) region that contains 14 genes and that is associated with the normal production of spermatozoa [38]. The AZF region is divided in 3 parts: AZFa, AZFb and AZFc and deletions of different parts of this region lead to different degrees of infertility, ranging from azoospermia to normozoospermia [39] (See Figure 1). For example, deletions in the AZFa region cover the two most important genes on that region, USP9Y and DBY, and cause Sertoli cell-only syndrome, in which there is a complete lack of sperm production, whereas deletions in the AZFb region can arrest spermatogenesis at the primary spermatocyte stage [40]. Most men with Yq microdeletions require the use of ICSI during their fertility treatments. It is important to note that male offspring of men with Y microdeletions will carry, unequivocally, the same microdeletion as the father, increasing the risk of presenting different levels of aneuploidies [36]. 

Gene mutations are considered to be any permanent change in the nucleotide sequence. Gene point mutations may involve the substitution, addition or deletion of single or multiple nucleotides. Some gene mutations are closely associated with male infertility due to physical or physiological alterations [41,42]. Mutations in the cystic fibrosis transmembrane conductance regulator cause cystic fibrosis and congenital bilateral absence of the vas deferens, thus causing obstructive azoospermia. Men with this problem can use ICSI as treatment, provided that the female does not carry the mutation as well [43,44]. 

The androgen receptor gene plays an important role in spermatogenesis. Mutations in this gene cause a variety of defects known as androgen insensitivity syndrome and are related to different grades of infertility, especially with asteno- and oligo-zoospermia [45]. 

Cryptorchidism is a condition in which the testes do not descend properly into the scrotum. If not treated, it may cause infertility due to increased scrotal temperature. There are two genes whose mutations have been linked with cryptorchidism, insulin like factor 3, a member of the relaxin-like hormone family produced by the Leydig cells, and its receptor, leucine-rich-repeat-containing G protein coupled receptor 8 [46].

#### 2.3.2. Genetics and Female Infertility

The most common genome rearrangement found in infertile women is 45X Turner´s syndrome. Women with this syndrome usually lack secondary sexual characteristics and have an abnormally small uterus, which explains why complete pregnancy is rare in this group [47]. Another chromosomal abnormality that can appear in women is the 47XXX syndrome. Although women with this syndrome are phenotypically normal, some studies have related this syndrome with premature ovarian failure [27]. Curiously, women with this syndrome do produce normal oocytes with a single X, which does not increase the risk of producing chromosomally abnormal offspring [48].

Sex-autosome translocations in the female are usually problematic as they are associated with a non-random X inactivation. Usually, the derivative X: Autosome chromosome (Xt) is the one that remains active after the X silencing, while the normal X chromosome (Xn) is inactivated. This preferential inactivation ensures that the autosome region present in the Xt remains active, as its inactivation would produce monosomy in the implicated autosomal region, which is lethal. This type of balanced translocation may be associated with gonadal dysgenesis and a 50% female infertility rate, although carriers are phenotypically normal [49].

X chromosome microdeletions/deletions in the X chromosome have been associated with a variety of female infertility depending both on the arm affected and the position of the deletion within it (Figure 1). 

Although gene mutations and polymorphisms associated with female infertility share similarities with those reported for male infertility, their incidence in the female is typically lower, due to the distinctive meiotic processes associated with the oocyte that include a long period of meiotic arrest and low number of gametes produced. Nevertheless, there are some gene mutations that have been demonstrated to cause female infertility; for example, mutations in the gene *HOXA13* affect uterine development and are related with recurrent pregnancy loss [50], while approximately two-thirds of women with mutations in the *GALT* gene have premature ovarian failure [51]. Moreover, it is important to consider that in both males and females, mutations in genes involved in hormonal regulation of gamete development are also related with different degrees of infertility [52].

## 3. DNA Damage in Reproductive Cells

### 3.1. Origin of DNA Damage in Reproductive Cells

**Figure 2:**
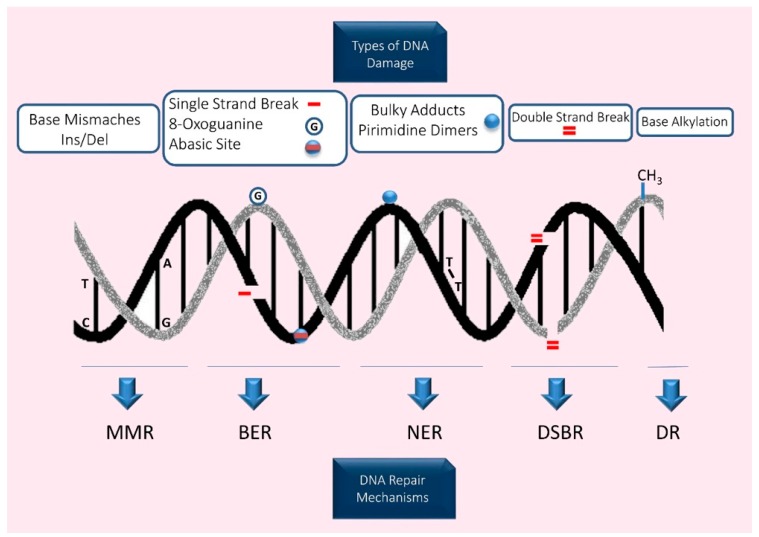
Diagrammatic representation of the different types of DNA damage and the DNA repair mechanisms involved in their reparation. MMR—MisMatch Repair; BER—Base Excision Repair; NER—Nucleotide Excision Repair; DSBR—DNA double Strand Break Repair and DR—Direct Reversal.

Different types of DNA lesions can be observed in all living cells and the gametes are no exception (summarised in Figure 2). 

For spermatozoa, the presence of sperm DNA damage in the ejaculate originates from three primary mechanisms (i) defective chromatin condensation during spermiogenesis, which is related to an inappropriate protamination and insufficient chromatin packaging [53]; (ii) the incidence of abortive apoptotic processes, as in mature spermatozoa, apoptosis cannot be completed due to the presence of the nucleus and mitochondria in different compartments [54]; and (iii) the incidence of oxidative stress as a result of the imbalance between reactive oxygen species production and the antioxidant capacity of the reproductive system to compensate adverse effects [55]. These mechanisms may be influenced by various parameters such as the age or the abstinence period of the male but can also be triggered by other situations such as exposure to stressful environmental factors (chemicals or radiation) or associated with pathological conditions (microorganism-mediated infections, cancer, varicocele or high temperatures). It is now well established that the proportion of sperm cells containing damaged DNA is higher in infertile males than in fertile controls [56]; that males with reduced semen quality are more likely to present with a higher percentage of sperm containing damaged DNA molecules than males with normal semen parameters [57]; and that fertilization mediated by a spermatozoon with damaged DNA can consequently have an adverse effect on embryo quality and development, blastocyst formation and the rate of pregnancy [58].

The susceptibility of the oocyte to DNA damage is less documented than in the spermatozoon, perhaps in part due to the difficulty of obtaining oocytes for research purposes. However, it is accepted that there are specific periods when the oocyte is more sensitive to external agents, and consequently there is a higher risk of DNA damage occurring. Oocytes are especially sensitive to DNA damage in the periods when they are dividing. Hence, this occurs during the fetal stage before they are arrested in prophase I and in mature life when they resume meiosis during the pre-ovulatory stage of the menstrual cycle [59]. 

Moreover, both types of gametes are also susceptible to iatrogenic DNA damage due to their manipulation during assisted reproduction treatments. As an example, both gametes produce higher levels of reactive oxygen species when they are exposed to the lights and fumes of the incubators, whereas spermatozoa are highly stressed when they are centrifuged [60]. This situation is especially problematic, as during assisted reproduction procedures, seminal plasma and follicular fluids are retrieved, thus depriving the reproductive cells of some of the natural occurring biological protective systems present in these biofluids such as the reactive oxygen species (ROS) neutralizing systems [60]. Although cryopreservation procedures of reproductive cells are useful to preserve fertility before cancer therapy or surgical infertility treatments in humans, they also pose another potential source of DNA damage for both gametes. The cause of this damage may not necessarily be associated with the freeze-thaw procedure itself but the oxidative damage caused by the post-thaw breakdown of the cell membrane and exposure to toxic metabolic products [61].

### 3.2. Defective Protamination and DNA Damage

As noted earlier, protamination is the process that takes place during the last phases of spermatogenesis, during which approximately 80% of the original histones are replaced by protamines in order to achieve a higher level of compactness in the sperm nucleus. Although advantageous for the sperm cell, the process of DNA protamination can also have detrimental effects on sperm quality, as errors in the replacement process can be associated with the production of damaged sperm DNA [62,63]. On the one hand, when histones are substituted by protamines, temporal breaks occur in the DNA due to topoisomerase II activity, which relaxes the DNA structure. In addition, if these temporal breaks are not repaired properly before the end of spermiogenesis, they will subsequently appear in the mature spermatozoa as fragmented DNA.

Alternatively, both the quantity of histones that are replaced by protamines (PRM) and the proportion of PRM1/PRM2 added are typically consistent for each species, so that if the proportion is changed, the DNA is likely to be poorly packaged and more susceptible to be affected by exogenous agents. For example, in humans the ratio between PRM1 and PRM2 is approximately 1 and several studies have shown that changes in this ratio may be related to male infertility [64,65,66]. It is possible that, as PRM2 contains fewer cysteine residues than PRM1, it therefore produces fewer disulfide bridges, leaving the DNA slightly more exposed to adverse effects of external agents. In addition, abnormal protamine ratios have also been related to male infertility through aberrant genomic imprinting [67].

### 3.3. Abortive Apoptosis and DNA Damage

During spermatogenesis, Sertoli cells select which germ cells pass from mitosis to meiosis. As a consequence of this screening procedure about a 60% of these germ cells are marked to be eliminated via apoptosis. However, varying percentages of these marked cells undergo abortive apoptotic processes in which their DNA is partially fragmented but they still maintain their capacity to differentiate into mature and even functional spermatozoa [68]. As mature spermatozoa are not capable of completing apoptosis, due to the nucleous and the mitochondria being in different compartments, the spermatozoa resulting from abortive apoptosis processes will appear in the ejaculate as sperm with high levels of fragmented DNA, even if they have a normal morphology [69].

### 3.4. Oxidative Stress and DNA Damage

ROS are molecules containing incompletely reduced oxygen atoms that are capable of reacting with almost all biomolecules [70]. These compounds are originated as a byproduct of the metabolism of oxygen during cell reactions and are indeed needed at low concentrations for some normal physiological functions. However, when the rate of ROS generation exceeds the neutralizing ability of the cellular antioxidant defense system, they can have detrimental effects inducing the inhibition/activation of enzymes, lipid peroxidation and DNA damage. Of four bases, guanine is the most susceptible to oxidation. The major oxidized form of guanine is 8-oxoG, which is endogenously generated by ROS, constitutively exists in DNA and is known to cause G to T and A to C transversion mutation during DNA replication. Ohno et al. [71] generated triple knockout (TOY-KO) mice and they concluded that 8-oxoG is the causative molecule for spontaneous and inheritable mutations of the germ lineage cells. A substantial number of studies support the notion that antioxidant supplementation involving melatonin, L-carnitine, selenium and N-acetyl-cysteine, both orally or in the ART culture media, is able to suppress or reduce oxidative stress and improve sperm and oocyte quality, leading to increased pregnancy rates [72].

#### 3.4.1. Oxidative Stress in the Spermatozoa

It is widely known that a physiological level of ROS in semen is necessary for basic sperm functions such as sperm capacitation, sperm motility, acrosome reaction and sperm-oocyte fusion [73,74]. ROS generation in the spermatozoon results from the activity of two enzymes; a NADPH oxidase located in the plasma membrane and a NADH dependent mitochondrial oxido-reductase. It is the NADH-dependent mitochondrial oxido-reductase that appears to be the main source of ROS in sperm cells, as the sperm midpiece is rich in mitochondria because they need a continuous supply of energy to provide motility.

However, spermatozoa are also affected by ROS present in the seminal plasma, which can have an endogenous or an exogenous origin [75]. Both leukocytes and immature spermatozoa are considered as the principal endogenous sources of ROS generation in the ejaculate while radiations and toxins are considered as external sources. Normally, ROS present in seminal plasma are neutralized by antioxidant systems that maintain ROS at a stable low concentration. However, under certain circumstances, this equilibrium can be disrupted and oxidative stress manifests resulting in lipid peroxidation, reduced membrane fluidity and DNA damage [75]. Spermatozoa are highly susceptible to ROS-induced damage due to the rich composition of polyunsaturated fatty acids in their plasma membrane and their reduced capacity for repair [76,77]. Several studies have confirmed that excessive levels of ROS in seminal plasma directly and/or indirectly lead to sperm DNA damage, abnormal semen parameters, impaired sperm function, and even infertility [78]. 

There are several natural antioxidant systems in seminal plasma which help to maintain the ROS concentration in balance. Firstly, there is an enzymatic antioxidant system that includes enzymes such as catalase, superoxide dismutase, glutathione peroxidase [79]. Secondly, seminal plasma is rich in antioxidant non-enzymatic compounds (for example vitamins C and E, carotenoids, lactoferrin or coenzyme Q10). Finally, it has been recently discovered that prostasomes present in seminal plasma decrease the release of superoxide radical by the leucocytes thus reducing oxidative stress [80].

#### 3.4.2. Oxidative Stress in the Oocyte 

The oocyte is exposed to different sources of ROS in the ovary. Endothelial cells, parenchymal steroidogenic cells and phagocytic macrophages produce ROS in the ovary [81] and this ROS is needed at moderate controlled concentrations for normal reproductive functions such as folliculogenesis, oocyte maturation, ovulation and corpus luteal function [82]. The process of oocyte maturation and ovulation can be seen as analogous to an inflammatory response, and consequently generates significant ROS output [83,84]. Moreover, ROS production has also been implicated in tubal function and cyclical endometrial changes [85]. Despite their exposure to normal ROS production, oocytes in general are considered to be reasonably resistant to oxidative stress during ovulation, a finding that seems to be closely related to the relatively high levels of antioxidants present in follicular fluids [86,87]. Enzymatic antioxidant defenses are also present in mammalian oocytes and embryos. Follicular and tubal fluids have also been reported to be endowed with enzymatic and non-enzymatic antioxidants [88]. 

However, under some conditions such as exposure to higher levels of oxidants or the presence of ovarian pathologies, the normal ROS equilibrium in the ovary may be disrupted and the concentration of ROS may increase higher than normal, reducing the fertilizing ability of the oocyte [89]. Excessive levels of ROS, if not properly mitigated, can lead to poor oocyte quality, under both in vivo and in vitro conditions [81,90]. Elevated levels of ROS in the oocyte can also alter oocyte cytoskeleton and microtubules, produce chromosomal scattering and aneuploidies [78]. In addition, elevated concentrations of ROS in the tubal and peritoneal microenvironment may affect the gametes and their capacity for interaction and syngamy in the Fallopian tube. Moreover, increased concentrations of ROS have been related to specific female pathologies producing infertility such us endometriosis [91].

### 3.5. Single-Stranded Breaks versus Double-Stranded Breaks

There are two different lesions that need to be considered when discussing sperm DNA fragmentation; single-stranded breaks (SSBs) and double-stranded breaks (DSBs). There are also different strategies that can be used to assess and differentiate these phenomena. One approach is the single-cell electrophoresis assay, which is commonly known as the comet assay [92,93]. This is a relatively simple method for assessing DNA strand breaks in eukaryotic cells in which chromosomes and DNA are detached using a controlled electrophoretic field. In general, it is assumed that the comet assay targets DSBs, but the alkaline comet assay can be used to detect and differentiate both DSBs and SSBs; using an extra step in the comet assay. It is possible to assess the simultaneous presence of DSBs and SSBs in a single cell using a two-dimensional or two-tailed comet assay [94].

The appropriate identification of such lesions is diagnostic as each type of break can be associated with the presence of a specific stressor [95]. For example, the presence of SSBs in the spermatozoa has been associated with oxidative stress or with the action of endogenous or exogenous DNA nucleases in the ejaculate, while the presence of DSBs has been associated with a defective repair of the temporal breaks produced during chromatin remodeling [96]. The type and complexity of DNA lesions may also influence embryonic development [97] because low levels of SSBs are easily repaired by the oocyte, while a large proportion of DSBs would typically exceed the oocyte’s repair capacity. In general, the presence of DSBs in sperm DNA is concomitant with delayed paternal DNA replication, paternal DNA degradation, and the subsequent arrest of embryo development [98]. Moreover, there is a higher risk that DSB DNA lesions will be mis-repaired when compared to SSBs, leading to detrimental mutations and infertility [99].

### 3.6. Susceptibility to De Novo Mutations

De novo mutations (DNMs) are novel genetic changes that are present in the genome of an individual but not in the genome of the somatic cells of its parents. These mutations can appear during gametogenesis, post-zygotically or during the postnatal life of the individual, but only those present in the germ cells will pass to the next generation [100]. DNMs include single nucleotide variants (SNVs), small insertions or deletions (indels <1000 bp) and large copy-number variants (CNVs), and can originate through different mechanistic failures such us mistakes during DNA replication, the effect of endogenous or exogenous mutagens or errors when repairing DNA damage [101]. As an example, it has been proposed that 8-oxoG is a causative molecule for spontaneous and inheritable SNV mutations of the germ lineage cells, especially for G to T changes [71]. The incidence of DNMs varies depending on the type of DNM; it has been estimated that the number of DNMs appearing per generation is 74 for SNVs, 3 for indels and 0.02 for CNVs [102]. More recently, the study of DNMs has gained increasing attention due to the rapid development of sequencing technologies. Various studies of parent-offspring trios have concluded that DNMs are a major cause of severe early-onset diseases such as developmental disorders, autism and intellectual disability [103,104]. Moreover, DNMs play an important role in pediatric diseases such as congenital heart defects [105] and also seem to associate with late-onset neurological and psychiatric disorders such as Parkinson´s disease, schizophrenia and bipolar disorder [106,107].

The incidence of DNMs varies between the spermatozoon and the oocyte as a result of the differential development of spermatogonia and oogonia into mature gametes. Studies comparing somatic and germline mutation rates have concluded that the mutation frequency is higher in the former [108]. It has been estimated that the mutation rate is similar in both sexes, both during the formation of the PGCs (0.2–0.6 mutations per haploid genome per cell division) and during the expansion of the PGCs to form the populations of spermatogonia and oogonia (0.5–0.7 mutations per haploid genome per cell division) [109]. However, in the later steps of gamete production, the situation differs substantially. After PGC expansion, oocytes only undergo one additional round of DNA replication while some spermatogonia (stem cells) literally undergo hundreds of rounds of DNA replication during the life time of the adult male. This situation causes a progressive accumulation of mutations in the spermatogonial genome due to the occurrence of errors during DNA replication [110]. It has been estimated that each additional year in paternal age at the time of conception adds 1–3 DNMs to the genome of the offspring, whereas in contrast, each additional year of maternal age adds only 0.24 DNMs [111]. Furthermore, not only does the DNM rate vary between oocytes and spermatozoa but also the type and location of the DNMs vary [112,113]. Non-recurrent de novo SNVs and CNVs show a strong paternal bias and age effect and have been correlated with errors occurring during the mitotic amplification of spermatogonia. In contrast, recurrent de novo CNVs show a strong maternal bias and tendency to appear in specific loci with higher recombination rates [113]. Recurrent structural variants often result from NAHR (non-allelic homologous recombination), also known as ectopic homologous recombination, that occurs during meiosis and causes genomic disorders resulting from crossovers between low-copy repeats located in the same chromosome or homologous chromosome. Non-recurrent structural variants can be characterized by simple blunt ends or microhomologies, such features indicate that are formed by other mechanism distinct from NAHR, such as NHEJ (non-homologous end joining) and RBMs (replication-based mechanisms) [114]. All of these mutations may contribute to an increased probability of parental low-level mosaicism. Germline mosaicism can alter the recurrent risk for future pregnancies [115].

Finally, it is also worth noting that not only is there a paternal bias for de novo gene mutations, but a similar bias has been reported in many cases of de novo structural chromosome aberrations in agreement with the susceptibility of post meiotic male germ cells to irreparable DNA damage [116,117].

## 4. DNA Repair in the Reproductive Cells

### 4.1. DNA Repair Mechanisms during Gametogenesis

**Figure 3:**
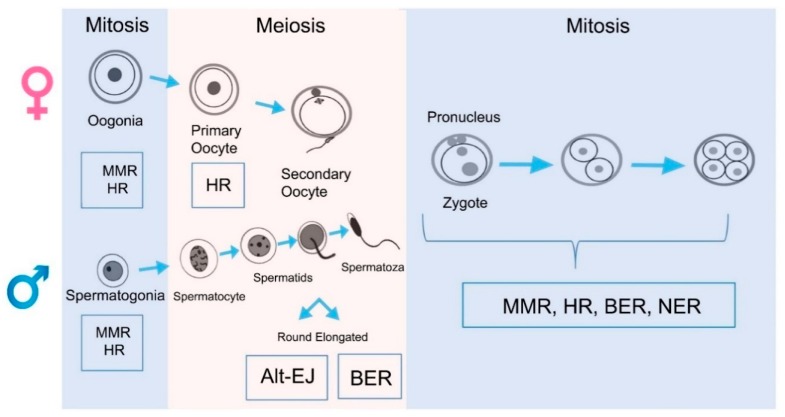
The primary DNA repair mechanisms occurring at the different stages of gamete and embryo production.

Gametogenesis in mammals involves a period where cell numbers are amplified, meiosis is completed, and haploid cells are morphologically and structurally transformed into sperm (spermiogenesis) or oocytes (oogenesis). Consequently, a complex balance of genome stability and instability is necessary, controlled by the interaction of several DNA repair mechanisms. In germ cells, there are several levels of defenses that avoid the production and persistence of DNA damage, such as base mismatches, SSB and DSB, bulky adducts, etc. Reproductive cells have an array of DNA repair pathways which include (i) nucleotide excision repair (NER), (ii) mismatch repair (MMR), (iii) base excision repair (BER), (iv) homologous recombination (HR), and (v) non-homologous end joining (NHEJ) (see summary in Figure 2 and Figure 3). 

NER is the DNA repair pathway that corrects a wide variety of helix-distorting DNA lesions and crosslinks primarily caused by environmental agents such us ultraviolet UV light [118]. MMR eliminates DNA mismatches produced from recombination between imperfectly matched sequences or from errors during DNA replication. The MMR system can also act in the repair of oxidative damage [119] as well as in the maintenance of repeated sequences [120]. BER corrects for small DNA alterations that only affect one DNA strand and that do not distort the structure of the DNA helix such as the incorporation of uracil or oxidized bases induced by reactive oxygen species or the presence of SSBs [121,122]. The lesion is removed and the complementary strand is used as a guide to fill in the gap. HR is a process that takes place during meiosis but is also used to repair DSBs as well as inter-strand DNA crosslinks (mainly produced by ionizing radiation) [123]. NHEJ repairs DSBs without using the homologous sequence as a template and thus can cause insertions and deletions. DNA direct repair mechanisms, photolyase-, alkyltransferase-, and dioxygenase- mediated repair processes, provide cells with simple yet efficient solutions to reverse covalent DNA adducts [124]. While these DNA repair mechanisms are active in practically all somatic cell types, as well as in the germ cells [125], mature sperm and oocytes show a differential organization of their repair mechanisms. 

### 4.2. DNA Repair Mechanisms in Male Germ Cells and Spermatozoa

Spermatogenesis is a complex process that produces spermatozoa, which are unique in cell morphology, chromatin structure and function. Spermatogenesis can be divided into three sequential steps: (i) mitotic proliferation, (ii) meiotic recombination and (iii) cytodifferentiation of spermatids [1]. Mitotic proliferation results in the production of large numbers of spermatozoa over the reproductive lifetime of the organism; mismatches produced during this step are repaired via the MMR pathway. Meiotic recombination and chromosome segregation produces genetically diverse haploid gametes. In terms of recombination, the two major pathways of HR and NHEJ appear to have divided their responsibilities based on the stage of the cell cycle and the nature of the DNA break. In particular, HR mainly operates during S phase and on replication-derived one-ended DSBs to faithfully resolve the damage, whereas the more error-prone NHEJ process functions primarily during G1 and on frank, juxtaposed two-ended DSBs [126,127]. It has been suggested that programmed DNA DSBs during meiosis are mainly repaired by HR with high fidelity. Repair of these breaks is tightly controlled to favor HR; the only repair pathway that can form crossovers. Cytodifferentiation of spermatids is a complex remodeling of the haploid genome which involves replacing the majority of histones with protamines. DNA compaction, which is an outcome of this process is achieved by transient formation of SSBs and DSBs in the sperm DNA [128]. Spermatids resolve exogenous and programmed DSBs using the alternative NHEJ pathway (Alt-EJ) [129] due to their haploid character and the absence of the main components of the classical NHEJ pathway. It is essential that these transition strand breaks are repaired during this step because the persistence of DNA breaks in the mature sperm can lead to increased sperm DNA fragmentation which is associated with subfertility [130].

Historically, mature spermatozoa were considered incapable for DNA damage repair because of the extreme compaction of their DNA and reduced transcriptional capacity [131]. However, it has recently been discovered that human spermatozoa possess a truncated but functional BER pathway containing only the OGG1 protein [132]. Being the first enzyme in the pathway, the presence of this enzyme is sufficient for the spermatozoa to detect and remove oxidized base adducts, specially 8-OHdG residues, a prevalent product of oxidative stress. Due to the rest of the pathway being truncated, the abasic site produced after the excision of 8-OHdG has to be subsequently repaired by the oocyte after fertilization and prior to the first round of cell division during early embryo development. 

At the end of spermiogenesis, disulfide cross-links are formed between protamines, while the spermatids pass through the epididymis. This process may be considered as an intrinsic screening mechanism directed at eliminating genetically defective sperm, as the higher the levels of DNA damage, the lower disulfide cross-linking established, resulting in lower quality spermatozoa with a reduced capacity to fertilize the oocyte or producing a viable embryo [133]. 

### 4.3. DNA Repair Mechanisms in the Oocyte

Oocytes are one of the most long-lived cells in mammalian species [118]. Essentially, oogenesis can be divided into three phases [134]: firstly, PGCs initiate their differentiation into female germ cells (oogonia) in the early post-implantation embryo; secondly, oogonia divide through mitosis and enter meiosis I until they stop developing at the diplotene stage, in prophase I and thirdly, oocytes complete the first division of meiosis I during ovulation. The integrity of the oocyte genome is thus affected mainly by two processes: (i) the meiotic recombination during the fetal period, and (ii) the long postnatal period of meiotic arrest (dictyate stage) before meiotic division. 

Physiological DSBs are produced in association with meiotic recombination during the fetal period; however, this damage is generally repaired at the end of the meiotic prophase I by the oocyte through HR [135]. Failure to repair DNA damage caused by recombination operates meiotic checkpoints and activates apoptosis [136]. With respect to the prolonged postnatal period of meiotic arrest prior to meiotic division, the oocyte DNA is subjected to a wide range of potential damage that can increase problems of female fertility [137,138]. Several studies provide strong evidence that oocytes, from primordial follicles stage to that of MII, have the capacity to repair damaged DNA and maintain genome integrity [139]. During oogenesis, genes related with DNA repair are expressed at high levels and their mRNAs and proteins are accumulated inside the oocyte cytoplasm [140]. Transcripts from all DNA repair pathways including direct lesion reversal, BER, MMR, NER, HR and NHEJ are represented in mouse, monkey and human MII oocytes and embryos [140,141,142,143]. These transcripts and proteins play a role during fertilization to address changes in chromatin remodeling and maintain chromatin integrity and are also used in the zygote until the embryo genome becomes active and it can transcribe its own DNA repair genes [141]. Recently Martin et al. [144] provided the first evidence that the MII oocyte has the potential to DNA repair via NHEJ in mice. However, a RNA-seq analysis suggested that there may be species differences in the ability of GV (germinal vesical stage) and MII oocytes to undertake DNA repair [145]. In this study, the overall expression patterns of genes involved in the repair of DNA double strand breaks were different between primates and mouse. Based on these data, it was proposed that rodent oocytes have a superior DNA repair competence to that of primates [145]. DNA repair efficiency in the oocyte also decreases with maternal age as a consequence of a reduction in the mRNA levels for the DNA repair genes [146].

### 4.4. DNA Repair Mechanisms in the Zygote

When male and female pronuclei are both observed within the ooplasm, the oocyte is characterized as a development stage known as ootid; following syngamy (fusion of the paternal and maternal DNA) a single cell embryo or zygote results. During this stage, there are three main processes related to the DNA that are taking place; (i) chromosomes initially exist separately as distinct maternal and paternal pronuclei, (ii) remodeling of chromatin structure with active demethylation of paternal DNA versus passive demethylation of maternal DNA [147] and (iii) reparation of SSBs and DSBs in the paternal DNA [148]. DNA repair in the zygote is considered a maternal trait because until embryonic genome activation (EGA) occurs (4-cell stage in humans), zygote development is supported by maternal transcripts and proteins [149].

As mature spermatozoa have reduced DNA repair capacity, some DNA lesions will inevitably remain in the sperm DNA and will need to be removed when gametes are joined in the zygote. The impact of sperm DNA damage after fertilization depends on the balance between the amount and/or type of DNA damage present at fertilization and the capacity of the fertilized oocyte to repair the sperm DNA molecule. It has been suggested that the oocyte has the capacity to repair sperm DNA damage when the level of sperm DNA damage is less than 8% [150,151]. Higher levels of sperm DNA damage are associated with a failure to reach the blastocysts phase [152] and embryonic loss between the EGA and the blastocyst stages [153,154]. This phenomenon is known as a “late effect” from paternal DNA damage [155].

## 5. Conclusions

The oocyte’s capacity to repair sperm DNA damage in the zygote stage also depends on the type of sperm DNA damage. SSBs and abasic sites that remain from the incomplete repair of SSBs in the mature spermatozoa can easily be repaired as the oocyte has the BER route. However, it is interesting to note that the expression of the OGG1 enzyme in the oocyte at this time is also very low [155]. The fortuitous complementarity of the sperm and oocyte has been regarded by some authors as a sophisticated mechanism to check the compatibility between the oocyte and the fertilizing spermatozoa as both need to participate to repair oxidative DNA damage [156]. In contrast to SSB repair, DSB repair in the zygote is carried out using NHEJ and HR. These pathways are not equally important during cell cycle. The balance between DSB repair pathways depend on the cell type and developmental stage, making HR relatively more important in first embryonic stages [157]. This is because DSBs produced by replication stalling are preferably repaired by HR [158]. However, in the zygotic stage, the NHEJ mechanism play an important role in repair of sperm DSBs [159]. 

Although both sperm and oocyte originate from PGCs in the human epiblast, they take on very different developmental pathways during early embryogenesis. Some spermatogonia remain as stem cells in the seminiferous epithelium of the testis to undergo multiple divisions during the lifetime of the adult male, resulting in billions of mature sperm cells and, therefore, many opportunities for DNA copy errors to occur. The mature spermatozoon is considered to be the most differentiated of cells and possesses a nuclear organization once it leaves the epididymis that is fundamentally different to the rest of the soma. By contrast, oogonia form a resident population of primary oocytes that remain in meiotic arrest until puberty, after which typically, only one oocyte is able to complete maturation within each menstrual cycle. Consequently, the oocyte may be considered as a form of “quasi-sedentary gamete” whose morphological and functional organization is more closely akin to that of somatic cells than of spermatozoa.

The fusion of dissimilar gametes resulting in the formation of a normal and viable embryo is known as anisogamy and provides the phenotypic variation on which natural selection may operate being, therefore, the basis of evolutionary potential. However, the developmental differences in gametogenesis have important consequences for their DNA packaging that make both cell types differentially susceptible to the effect of stressful environments, in terms of both normal reproductive physiology and/or exposure to adverse external agents, which then makes them prone to suffer different types of DNA damage. While both gametes have the capacity to potentially repair the DNA molecule, this capacity is substantially compromised in the sperm cell. Of particular interest in the DNA repair mechanism, is the ability of the oocyte to repair DNA damage post-fertilisation and the notion that some repair processes require the synergistic complementary collaboration of both gametes. 

Male and female gametes must be of high quality to produce high-quality embryos, and their intact genomes ensure faithful transmission of genetic information to the next generation. In germ cells, there are several levels of defenses that avoid the production and persistence of DNA damage. Detoxifying peptides and proteins and antioxidants such as vitamins E and C help prevent DNA damage. However, this does not imply that germ line cells are a safe haven for DNA because mammalian germ cells can contain several types of DNA damage. DNA damage repair involves the cooperation between male and female gametes prior to the initiation of embryo development. Therefore, even if the fertilizing spermatozoon carries DNA damage in its genome, the oocyte could repair it and, therefore, it would be of no consequence to the embryo and to fetal development. However, it is extremely difficult to establish whether the oocyte is capable of repairing this damage. In addition, DNA fragmentation tests currently available cannot provide information concerning the ‘‘repairability’’ of DNA damage.

In developed countries, couples affected with infertility resort to ART in order to procure a family. Regrettably, it is estimated that approximately half of ART procedures are unsuccessful and it is likely that a large proportion of this failure are associated with defective gametes incompatible with fertilization and/or embryonic development. Moreover, with the increasing use of techniques such as intracytoplasmic sperm injection, an ART procedure in which the spermatozoa is selected and directly inserted into the ooplasm by the embryologist, several of the natural gamete selection steps do not take place, so that the risk of using paternal genomes that are defective or incompatible with the maternal is likely to increase. Consequently, a better knowledge of the genomic organization of the oocyte, spermatozoon and early embryo is needed in order to obtain a better understanding of the causes of ART failure, as well as to improve the current available treatments. Moreover, it is also vital to get a deeper knowledge of the differences in genome organization, susceptibility to DNA damage and DNA repair mechanisms between the spermatozoa and the oocyte, as these differences are likely to be critical for gamete compatibility, embryo development and even to elucidate the origin of some of the major early onset diseases.

## Abbreviations

Alt-EJ	Alternative NHEJ pathway
ART	Assisted reproduction technology
AZF	Azoospermia factor
BER	Base excision repair
CCRs	Complexchromosomerearrangements
CNVs	Copynumbervariants
DNMs	De novo mutations
DNA	Deoxyribonucleic acid
DSBs	Double stranded breaks
HR	Homologous recombination
ICSI	Intracytoplasmic sperm injection
LINES	Long interspersed nuclear elements
LTR	Long terminal repeats
MMR	Mismatch repair
NHEJ	Non homologous end joining
NER	Nucleotide excision repair
PGCs	Primordial germcells
PRM	Protamine
ROS	Reactive oxygen species
SINE	Short interspersed nuclear elements
SNVs	Single nucleotide variants
SSBs	Single stranded breaks
TEL-DNA	Telomeric DNA
TNP	Transition nuclear proteins
